# Functional Role of Dendritic Cell Subsets in Cancer Progression and Clinical Implications

**DOI:** 10.3390/ijms21113930

**Published:** 2020-05-30

**Authors:** Annalisa Del Prete, Francesca Sozio, Ilaria Barbazza, Valentina Salvi, Laura Tiberio, Mattia Laffranchi, Angela Gismondi, Daniela Bosisio, Tiziana Schioppa, Silvano Sozzani

**Affiliations:** 1 Department of Molecular and Translational Medicine, University of Brescia, Viale Europa 11, 25123 Brescia, Italy; annalisa.delprete@unibs.it (A.D.P.); francesca.sozio@unibs.it (F.S.); i.barbazza@unibs.it (I.B.); valentina.salvi@unibs.it (V.S.); laura.tiberio@unibs.it (L.T.); mattia.laffranchi@unibs.it (M.L.); daniela.bosisio@unibs.it (D.B.); tiziana.schioppa@unibs.it (T.S.); 2 Humanitas Clinical and Research Center—IRCCS, Via Manzoni 56, 20089 Rozzano (MI), Italy; 3 Laboratory Affiliated to Istituto Pasteur Italia-Fondazione Cenci Bolognetti, Department of Molecular Medicine, Sapienza University of Rome, Viale Regina Elena 291, 00161 Rome, Italy; angela.gismondi@uniroma1.it

**Keywords:** dendritic cell subsets, cancer, tumor microenvironment, cancer immunotherapy, migration, cytokines, chemokines

## Abstract

Dendritic cells (DCs) constitute a complex network of cell subsets with common functions but also with many divergent aspects. All dendritic cell subsets share the ability to prime T cell response and to undergo a complex trafficking program related to their stage of maturation and function. For these reasons, dendritic cells are implicated in a large variety of both protective and detrimental immune responses, including a crucial role in promoting anti-tumor responses. Although cDC1s are the most potent subset in tumor antigen cross-presentation, they are not sufficient to induce full-strength anti-tumor cytotoxic T cell response and need close interaction and cooperativity with the other dendritic cell subsets, namely cDC2s and pDCs. This review will take into consideration different aspects of DC biology, including the functional role of dendritic cell subsets in both fostering and suppressing tumor growth, the mechanisms underlying their recruitment into the tumor microenvironment, as well as the prognostic value and the potentiality of dendritic cell therapeutic targeting. Understanding the specificity of dendritic cell subsets will allow to gain insights on role of these cells in pathological conditions and to design new selective promising therapeutic approaches.

## 1. Introduction 

Dendritic cells (DCs) represent the bridge between innate and adaptive immune responses [1]. They are specialized in antigen recognition and presentation and play a central role in the initiation of antigen-specific immunity as well as tolerance [2]. Activation and maturation of DCs depend on the signals present in the local microenvironment, which are recognized by surface and intracellular receptors able to sense Pathogen- and Damage-Associated Molecular Patterns (PAMPs and DAMPs) and cytokines [3]. DCs are a heterogeneous immune cell population with consistent functional plasticity and are distinguished in different subsets according to their ontogeny, phenotype, tissue distribution, and molecular signatures both in mice and humans [4,5,6].

DC subsets are classified as conventional DCs (cDCs), plasmacytoid DCs (pDCs), and monocyte-derived DCs (moDCs) [6,7]. Based on the repertoire of transcription factors that control their development, cDCs can be further divided into cDC1s, which are under the control of IRF8, ID2, and BATF3, and cDC2s, which develop under the control of IRF4, ID2, ZEB, and Notch2/KLF4 [8]. The two cDC subsets differ for both phenotypical and functional aspects. cDC1s (CD141/BDCA3^+^ in humans and CD8a^+^ or CD103^+^ in mouse) preferentially express the chemokine receptor XCR1 and the C-type lectin receptor DNGR-1/CLEC9A [9,10,11], and are specialized in cross-presentation of exogenous antigens on MHC-I to CD8^+^ T cells. cDC2s (CD1c^+^ in humans and CD11b^+^ in mouse) preferentially express the signal regulatory protein CD172a and are specialized in the presentation of antigens on MHC-II to CD4^+^ T cells [5]. PDCs are characterized by the expression of B220 and PDCA1 in mouse and CD123, BDCA2, and BDCA4 in humans, and are potent type I IFN producers [5,12]. MoDCs represent an additional subset that arise during inflammation. MoDCs develop from monocytes recruited to the inflammatory site and are absent under homeostatic conditions. MoDCs can promote CD4^+^ T cell polarization within inflammatory contexts [3,13]. Additional human DC subsets might exist, as suggested by advanced high-throughput analysis [14]. Further complexity in DC biology is related to different migratory properties and tissue localization of DC subsets [15,16]. Recruitment of specific DC subsets in pathological conditions, such as cancer, may represent a key step in the instruction of protective immune responses [17,18]. Indeed, different DC subsets play specific roles in antitumor immunity through the expression of costimulatory molecules and inflammatory cytokines and have the ability to activate specific T cell subsets [19]. At the same time, DCs can favor the formation of a tumor-promoting local microenvironment by secreting anti-inflammatory cytokines and expressing immune checkpoint molecules able to restrain T cell response [20].

In this review, we will discuss: (i) the specific role of DC subsets in fostering or suppressing tumor growth; (ii) the molecules responsible for the recruitment of DC subsets into tumor microenvironment (TME); (iii) the prognostic value of DC subsets in TME, and (iv) the potential therapeutic implications of targeting specific DC subsets in cancer immunotherapy.

## 2. Anti-Tumor Activity of DC Subsets 

An efficient T cell-mediated antitumor immune response requires cross-presentation of tumor-associated antigens by DCs [21]. cDC1s represent a subset specialized in cross-presenting tumor antigens on MHC-I molecules to CD8^+^ T lymphocytes for the generation of efficient cytotoxic T cell (CTL)-mediated immunity (for a review, [22,23]). The critical role of cDC1s in antitumor immune defense was demonstrated by the genetic model of *Batf3* deficient mice, where cDC1 depletion led to the inability to reject transplantable immunogenic tumors [24,25,26] and to sustain immunotherapies based on adoptive T cell transfer or immune checkpoint inhibition [25,27,28]. Several molecules involved in membrane trafficking are required for efficient tumor antigen cross-presentation, such as the Soluble NSE Attachment Protein Receptor (SNARE) member Sec22b and the regulator of vesicular trafficking WDFY4. These molecules are also required for the control of tumor growth and for the efficacy of anti-PD1-based immunotherapies [29,30]. In addition to cross-presentation, other cDC1-associated molecules are necessary to promote anti-tumor immunity and tumor rejection [31]. For the initial priming of CD8^+^ CTLs tumor antigens must be delivered to tumor-draining lymph nodes by migratory CD103^+^ cDC1s in a CCR7-dependent manner [32]. Although resident CD8α^+^ cDC1s may also be involved, migratory CD103^+^ cDC1s have unique abilities in tumor-antigen cross presentation [27,32]. The expression of XCR1 is crucial for cDC1 functions, since it favors their localization in response to the ligand (XCL1) produced by CTLs and NK cells and the XCR1/XCL1 axis appears indispensable in the development of efficient cytotoxic immunity [33,34]. cDC1s in turn orchestrate local anti-tumor immunity, being the main producer of CXCL9 and CXCL10, two chemokines active on CXCR3^+^ effector T and NK cells [28,35]. Both chemokines are considered to be crucial also in the positioning of memory CD8^+^ T cells in cDC1-rich areas in order to promote local T cell restimulation [36,37]. Moreover, by locally producing high amounts of IL-12, cDC1s promote CTL and NK cell cytotoxicity and IFN-γ production [25,38,39,40]. As a positive feedback loop, IFN-γ boosts IL-12 production by cDC1s and potentiates cross-presentation [38,41]. By producing CCL5, NK cells can recruit circulating cDC1s to neighboring tissues and tumors [42]. Intratumor cDC1s represent a crucial source of Flt3L a factor that sustains the viability and functions of cDC1s within the TME and promotes their local differentiation from precursor cells [43]. cDC1s not only promote CTL expansion by MHC-I-mediated Ag presentation but also promote the generation of CD4^+^ Th1 cells through the presentation of antigens on MHC class II [44]. The antitumor functions of cDC1s may also be supported by pDCs [45]. pDCs are a major source of type I IFN, a potent activator of antigen cross-presentation and CD8^+^ T cell antitumor response [46,47]. T cell-mediated anti-tumor response may also be induced by cytosolic DNA from dying tumor cells through the activation of cGAS/STING-mediated type I IFN production [48]. In summary, the interaction of cDC1s with components of both innate and adaptive immunity represents an efficient and versatile system for CTL activation and antitumor functions.

The role of cDC2s in cancer immunology is apparently more limited compared to that of cDC1s. This is possibly due to the lack of selective membrane markers that allow the clear identification of these cells in pathological contexts and the availability of few preclinical studies. Even if cDC2s are in many aspects less efficient than cDC1s, such as in taking up tumor antigens, trafficking to draining lymph nodes, producing IL-12, and stimulating CD8^+^ T cells [25,27,28,32], these cells are very efficient in the presentation of MHC-II-associated tumor antigens to CD4^+^ T cells [49,50,51,52,53]. Activated CD4^+^ T cells contribute to antitumor immunity not only by concurring in CTL activation, but also through the production of IFN-γ that activates NK cells and macrophages, inhibits angiogenesis, regulates the generation of tumor stroma, and promotes direct cytolytic effects [54].

The cross-talk between T cells and DC subsets plays a crucial role at different levels. Maximal induction of the cytotoxic CD8^+^ CTL response requires not only cDC1s, but also involves cDC2, as shown by differential localization and spatiotemporal interactions of the two DC subsets in draining lymph nodes during viral infection [44]. A similar type of collaboration is also conceivable to happen in tumors [55,56]. During tumor growth, cDC2s were shown to be suppressed in their ability to induce differentiation of antitumor CD4^+^ T cells. Depletion of T regulatory (Treg) cells was shown to enhance their migration and ability to prime proinflammatory CD4^+^ T cells for IFN-γ production and tumor rejection [57]. Moreover, a role for tumor cDC2s in inducing activation of CD4^+^ T cells towards IL-17-producing T lymphocytes has also been described [49]. Th17 cells are apparently crucial for the efficacy of cDC2 vaccination because of their capacity to reprogram pro-tumoral macrophages and to reduce suppressive myeloid cells [49].

Human studies show some overlapping functions between cDC1s and cDC2s, such as IL-12 production and requirement of Flt3L for their development [58,59]. Similar to cDC1s, the number of circulating cDC2s is usually decreased in tumor patients [60]. Nevertheless, cDC2s were shown to be part of an immune signature in early lung adenocarcinoma lesions [61]. In breast cancer lesions, the expression of costimulatory molecules by cDC2s were differentially regulated in relation to cancer subtype, being higher in triple negative than in luminal breast cancers [62]. cDC2s do not express a unique gene signature. Indeed, cDC2 share a common signature with monocytes, with only a few genes selectively expressed, such as *CCL22*, a gene that encodes for a chemokine active on CCR4^+^ T cells [62]. In a different study, the gene *CD207* (that encodes for langerin) was identified as a specific marker for tumor-associated cDC2s, both in mouse and human lung cancers [63].

PDCs may favor antitumor immunity mostly through the production of IFN-α, an inhibitor of tumor cell proliferation, angiogenesis, and metastasis [64]. PDCs possess direct cytotoxic activity through the expression of TRAIL and Granzyme B [65,66]; this function was reported both in in vitro and in vivo experimental models [67,68]. TLR7-mediated production of type IFN I is essential for the regulation of TRAIL and Granzyme B secretion by pDCs via IFNAR1 signaling [65,66,67] and inhibition of this pathway by an anti-BDCA-2 moAb resulted in decreased TRAIL-mediated cytotoxic functions [65]. PDCs can exert also indirect antitumor effects through the CCR5-mediated recruitment of NK cells and the OX40L-mediated induction of IFN-γ [69]. In head and neck squamous carcinoma, a morphologically, functionally, and transcriptionally unique pDC subset expressing high levels of OX40 was described for being able to synergize with cDCs in generating potent tumor antigen specific CD8^+^ T cell responses [70].

**Figure 1:**
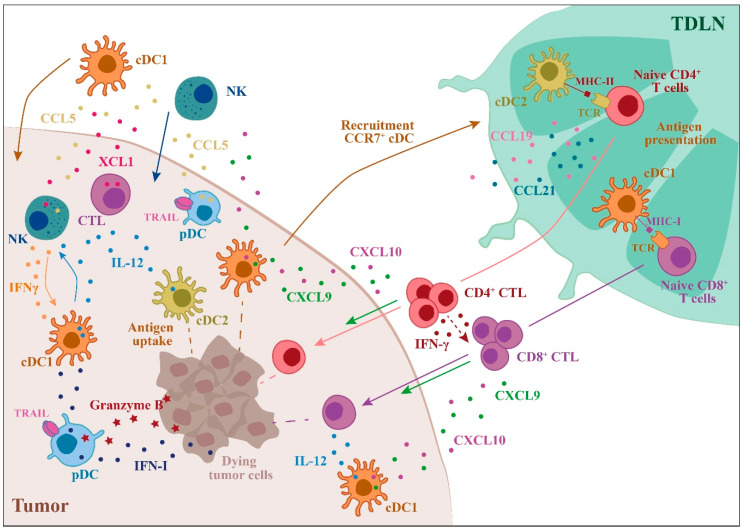
Role of dendritic cells (DC) subsets in the regulation of the anti-tumor immune response. The main events that involve DC subsets and contribute to a robust anti-tumor response are illustrated. The anti-tumor action of DC subsets starts with the uptake of tumor antigens followed by DC recruitment to the draining lymph nodes, where antigen presentation to T cells occurs. cDC1s are specialized in tumor antigen cross-presentation to CD8+ T cells, leading to tumor-specific cytotoxic T cell (CTL) differentiation, whereas cDC2s are the most efficient CD4^+^ T cell activators. In the tumor microenvironment (TME), DCs induce the recruitment and activation of NK cells and CTLs through the production of IL-12 and other chemokines/cytokines. Plasmacytoid DCs (PDCs) can kill tumor cells through the expression of TRAIL and Granzyme B (TDLN = tumor draining lymph node).

Because of the high degree of overlap with other myeloid cells, the relevance of moDCs in human tumors is unclear. MoDCs may have an important role in anti-tumor response promoting the proliferation of naïve CD8^+^ T cells [71]. In preclinical studies, moDCs were found to play a crucial role in mediating immune responses during chemotherapy, T cell adoptive therapy, and cell vaccination [72,73,74]. The main mechanisms exploited by DC subsets to perform efficient anti-tumor immune responses are summarized in Figure 1.

## 3. Tumor-Mediated Suppression of DC Functions

**Figure 2:**
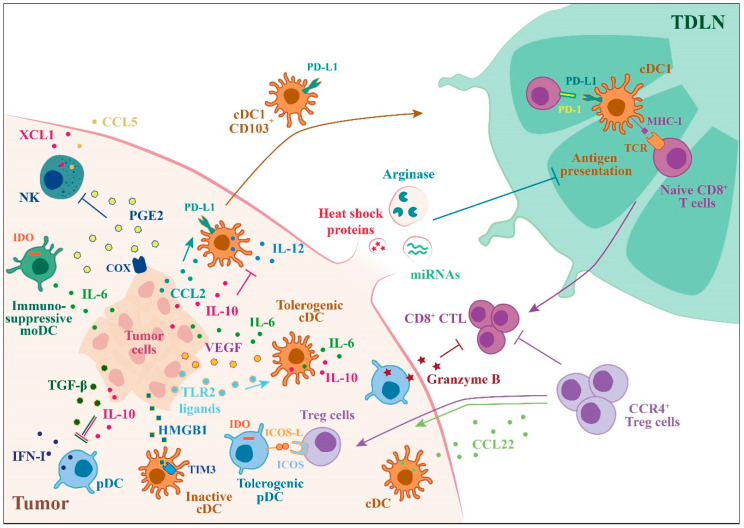
Tumor-mediated suppression of DC functions. Outline of the mechanisms by which tumors can alter DC functions switching their phenotype in immunosuppressive or tolerogenic, helping tumor growth and its escape from immune responses. Tumor cells secrete a variety of factors that can block the production of type I IFN (IFN-I) by pDCs or IL-12 by cDCs (IL-10, TGFβ) induce immunosuppressive moDCs (IL-6) and inhibit the recruitment of NK cells (PGE2). Tumor cells are also able to induce the expression of ICOS and PD-L1 by DCs creating an immunosuppressive microenvironment.

DCs can favor tumor growth and progression by promoting immune tolerance [75] (Figure 2). Within the TME, several soluble factors can upregulate transcriptional and metabolic pathways permissive for the generation of DC tolerogenic phenotype, such as IL-10, IL-6, PGE2, VEGF, and colony stimulating factor-1 (CSF-1). VEGF was one of the first identified factors produced by tumor cells described to inhibit DC functions, including differentiation from precursors, activation, and recruitment to the tumor site [76,77,78]. IL-10 production by tumor-associated macrophages can suppress the expression of the anti-tumor cytokine IL-12 by CD103^+^ cDC1s. IL-10 receptor blockade, in combination with CSF-1 inhibition, was shown to reduce metastatic burden and to improve the efficacy of paclitaxel therapy [39]. In addition, DCs may by themselves produce inhibitor factors. Tumor-derived TLR2 ligands were shown to be critical for the generation of immunosuppressive IL-6- and IL-10-producing DCs [79]. The Wnt/β-catenin activation in tumor cells has been variously implicated in the suppression of DC functions by paracrine IL-10 production and the downregulation of CCL4, a chemokine responsible for the recruitment of CD103^+^ cDC1s [28]. Within the TME, DCs are the main producers of CCL22, a chemokine that regulates the migration of CCR4^+^ Treg cells [80,81]. Treg-DCs interaction at the tumor site is critical for the local suppressive functions of Treg [82,83]. Tumor-intrinsic upregulation of COX activity and PGE2 production may be responsible for impaired NK cell recruitment and the consequent reduction of CCL5- and XCL1-mediated intratumoral cDC1 and CTL infiltration [42,84]. The enzyme indoleamine 2,3 dioxygenase (IDO) is upregulated in tumor-associated DCs. IDO-expressing DCs consume tryptophan, an essential aminoacid for effector T cell functions, promoting Treg differentiation [85]. Tumor-derived lactic acid was recently shown to impair DC functions in lung cancer [86]. The abnormal accumulation of lipids is a major mechanism involved in DC dysfunction, in particular in cross-presentation, as shown in several preclinical models and cancer patients [87,88]. In ovarian cancer, DCs engulfed with lipid droplets showed robust activation of the endoplasmic reticulum stress response factor XBP1. This pathway is responsible for triglyceride metabolism and accumulation of intracellular lipids, a mechanism that makes DCs unable to induce antitumor responses [89].

In addition to soluble factors, several membrane proteins concur in the suppression of DC functions. Production of the immunosuppressive chemokine CCL2 by stromal cells contributes to the generation of immunoregulatory DCs with decreased HLA-DR expression and upregulation of PD-L1 [90]. In mouse models of advanced ovarian cancer and hepatocellular carcinoma, PD-1 expression by tumor-infiltrating cDCs suppresses CD8^+^ T cell activity and decreases T cell infiltration [91,92]. In addition, PD-L1-expressing DCs can promote the expansion and function of Treg [93]. The inhibitory checkpoint receptor, TIM3, when expressed by cDCs infiltrating breast cancer, inhibits CTL recruitment through the downregulation of CXCL9 expression [94]. Furthermore, the release of HMGB1 by dying tumor cells was shown to compete with nucleic acid binding to the receptor TIM3 selectively expressed by intratumoral DCs with consequent inhibition of anti-tumor immune responses [95]. The expression of macrophage galactose N-acetyl-galactosamine specific lectin 2 (MGL2/CLEC10A) was described on an immunosuppressive subset of PD-L2^+^ DCs that accumulates in liver metastasis of pancreatic cancer as responsible for Treg development. Blocking PD-L2 or depletion of MGL2^+^ cells selectively activated CD8^+^ T cells and suppressed metastasis, suggesting that DCs use this pathway to inhibit CD8^+^ T-cell-mediated tumor immunity [96].

Recent evidence demonstrates a role for tumor-derived extracellular vesicles (EV) in promoting tumor progression and metastasis [97]. EVs derived from ovarian cancer were shown to deliver arginase-1 to DCs present in draining lymph nodes and to inhibit antigen-specific T-cell proliferation [98]. In another study, tumor-derived exosomes were reported to induce immune suppression through the delivery of heat shock proteins to DCs, leading to increased IL-6 production, which dramatically promoted tumor invasion by increasing signal transducer and activator of transcription 3 (STAT3)-dependent matrix metalloproteinases 9 transcription activity in tumor cells [99]. Moreover, tumor-derived exosomes can deliver different miRNA which play crucial roles in regulating DC functions [100].

In the TME, pDCs tend to be tolerogenic and favor tumor progression. Several studies have shown that tumor-associated pDCs are immature and have a diminished capacity to produce IFN-α, as originally shown in head and neck squamous carcinoma [101]. HMGB1 secretion during cervical carcinogenesis was described to be responsible for the decrease in IFN-α production and impairment of pDC functions [102]. PDCs can induce Treg cells through IDO or inducible T cell co-stimulator ligand (ICOSL). The presence of ICOSL-expressing pDCs correlates with breast cancer progression by a mechanism involving the induction of IL-10-producing Treg cells [103]. PDC-dependent, IL-10-producing, regulatory CD8^+^ T cells can suppress the generation of antigen-specific effector T cells by cDCs, in ovarian cancer [104,105]. In melanoma, pDCs, through OX40L and ICOSL, support tumor progression by promoting type 2 immune responses [106]. Recently, TGF-β was described as the main factor responsible for pDC immunosuppressive phenotype, being responsible for the inhibition IFN-α and MHC I expression following TLR9 activation [107]. In melanoma, IDO^+^ pDCs stimulation of CD4^+^CD25^+^FoxP3^+^ Tregs caused the upregulation of PDL-1 and PDL-2 in DCs, supporting a immunosuppressive microenvironment [108,109]. PDCs can favor tumor growth by promoting neoangiogenesis and producing high levels of IL-1α [110,111]. Finally, Granzyme B-secreting pDCs may play a regulatory role in immune evasion by affecting T cell proliferation [112].

Additionally, moDCs can be conditioned by TME to acquire an immunosuppressive, regulatory phenotype, as described in different models of ovarian cancer [113]. In the TME of many tumors (e.g., breast, colon cancer and leukemia) moDCs become efficient inducers of Treg and poor stimulators of allogeneic T cells [114,115]. Several products (such as IL-6, PGE2, ROS) from tumor cells and other immune cells can impair moDC differentiation and survival and block their antigen presenting functions, leading to a tumor-promoting phenotype [116,117].

## 4. Regulation of DC Subsets Migration in TME

**Figure 3:**
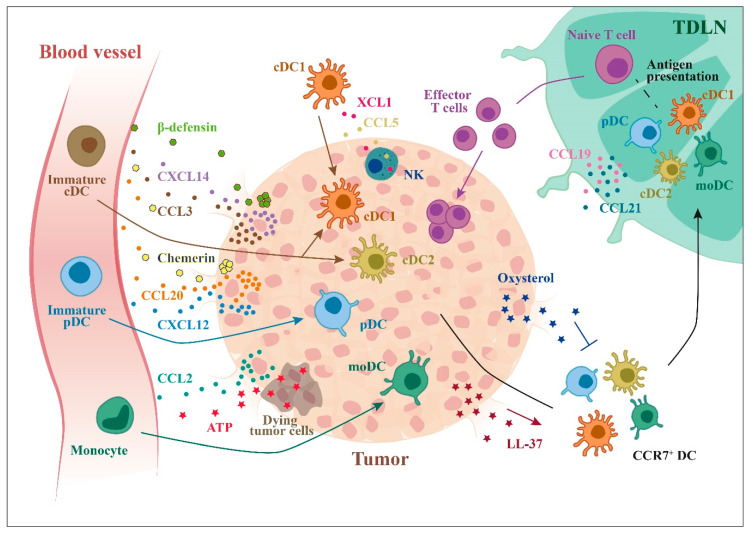
Recruitment of DCs to tumor microenvironment. Tumor cells can secrete many factors that can recruit different DC subsets: chemokines, such as CCL20, CXCL12, and CXCL14, and other molecules, such as ATP (from dying cells) or β-defensins. Tumor cells can also produce factors that inhibit the recruitment of CCR7^+^ DCs to lymph nodes (e.g., oxysterols).

Chemokines are master regulators of DC tissue distribution. DCs express a complex repertoire of chemokine receptors that are responsible for their trafficking between primary and secondary lymphoid organs and to peripheral tissues. Proper trafficking of DCs is required to promote T cell activation, proliferation, and survival [15,118]. Both cDC1s and cDC2s were shown to take up tumor antigens [25] and to migrate to tumor draining lymph nodes in a CCR7-dependent manner [32,49,119] (Figure 3).

Tumor infiltrating cDCs derive from a bone marrow precursor (pre-cDC) that migrate from blood to tumor to differentiate in proliferating cDCs. In different transplantable tumor models, this process was shown to be largely driven by the tumor expression of CCL3, a ligand of CCR1 and CCR5, two chemokine receptors expressed by pre-cDCs [120]. However, tumor moDCs may also develop and function independently of pre-cDC-derived DCs [74], as already differentiated circulating cDC1s may accumulate in the TME in response to the production of CCL5 and XCL1 by tumor-infiltrating NK cells, CD8^+^ T cells, and innate lymphoid cells [42]. While chemokines, such as CCL5, may also recruit cells that promote tumor growth, such as Treg and macrophages [121,122], XCL1 is a specific chemoattractant for cDC1s [123,124] and this chemokine could be exploited in cDC1-targeted therapies [125]. Many tumors also produce CCL20, as documented in primary breast carcinoma [126], renal carcinomas, and thyroid papillary carcinoma [127,128,129].

Infiltration of TME by cDCs is associated with favorable prognosis and response to immunotherapy [28,43]. Tumors with active intrinsic β-catenin pathway are characterized by reduced accumulation of cDC1s, leading to uncontrolled tumor growth [130]. In addition, tumor cell intrinsic factors may restrain cDC1 migration and usually, tumor infiltrating cDCs have immature phenotype. Indeed, CCL20 produced in TME preferentially recruits immature CCR6^+^ cDCs, with mature cDCs being confined to peritumoral areas [127,128,129,131]. Tumor-derived oxysterols were shown to inhibit DC trafficking to draining lymph nodes by downregulating CCR7 expression [132].

Proteins other than chemokines, such the antimicrobial inflammatory peptides β-defensins, are involved in the recruitment of CCR6^+^ DCs, as shown in ovarian and cervical cancers [78,133]. Other antimicrobial peptides, such as LL-37, a member of the cathelicidin family, may promote lymph node recruitment of cDC1s through the upregulation of CCR7 and XCR1, as described in a model of murine squamous cell carcinoma [134]. CRAMP (Cathelicidin-Related AntiMicrobial Peptide), the mouse homologue of LL-37, binds the formylpeptide receptor-2 (Fpr2), a receptor expressed by DCs [135]. Moreover, formylpeptide receptor 1 (FPR1) was described to be required for the correct localization of DCs in proximity of dying cells following anthracyclines treatment and eliciting antitumor T cell immunity [136]. Complement components are also involved in DC recruitment as described in colon cancer hepatic metastasis [137].

PDCs infiltrate both primary and metastatic tumors with the most studied tumor being melanoma. Melanoma cells produce CCL20 that can recruit immature circulating pDCs through CCR6 [138]. Mature pDCs are mainly localized in the peritumoral area of melanoma lesions with some of them also found in association with malignant cells, suggesting a possible role for pDCs in starting antitumor immune response [139]. Immature pDCs also express CXCR4, the receptor for CXCL12, a chemokine produced by tumor cells [140]. The CXCL12/CXCR4 axis is also involved in the recruitment of immature pDCs in ovarian cancer where high levels of CXCL12 are produced by epithelial tumor cells [141]. CXCL12 can act as a survival factor for tumor infiltrating pDCs [142]. Likewise, tissues of patients with head and neck squamous cell carcinoma showed pDC infiltration in response to CXCL12 [101]. The ability of the constitutive chemokine CXCL12 to induce pDC recruitment is controlled by the expression of CXCR3 ligands [143]. Finally, pDCs isolated from non-small cell lung cancer lesions were shown to migrate in response to CCL19 and CCL13 [144].

Circulating monocytes are recruited into tumor through the CCL2/CCR2 axis, as described in breast cancer [145]. Once in the TME, they can differentiate in macrophages or in moDCs [146]. Moreover, stromal primary cells derived from biopsies from prostate cancer patients contained high levels of CCL2, a monocyte chemoattractant. In the presence of stromal derived tumor-promoting factors, monocytes can differentiate in moDCs [147]. Chemotherapy-induced dying cells can release ATP, which recruits a DCs resembling inflammatory moDCs [72].

## 5. Prognostic Value of DC Subsets in TME

By using public datasets of gene expression profiles of whole tumor tissues, the presence of tumor infiltrating cDC1s has emerged to correlate with a better clinical outcome in a variety of different solid tumors. In breast cancer, independent studies have documented that elevated infiltration of cDC1s represents a positive prognostic value [25,38,42]. Similarly, a cDC1 transcriptomic signature was as predictive as a CTL signature of better survival [62]. The positive prognostic value of tumor cDC1 infiltration was apparently more important in triple negative breast cancer (TNBC) than in luminal cancer or other breast cancer subtypes [25,38,42]. A positive prognostic value for cDC1 infiltration was also reported for other solid tumors, such as head and neck squamous cell carcinomas, lung adenocarcinomas, and melanomas [32,43,148]. The analysis of a large cohort of breast primary tumors highlighted the selective role of cDC1-derived IFN-lamba1 in the generation of a microenvironment rich of Th1-cytokines and chemokines active on cytotoxic lymphocytes. Both IFN-lamba1 and its receptor were associated with favorable patient outcomes [149].

Tumor infiltrating cDCs show a dynamic distribution over time and can influence disease progression at different stages of tumor growth [150,151]. For instance, increased cDC infiltration was associated in early pancreatic ductal adenocarcinoma (PDAC) lesions with better anti-tumor immunity of CD8^+^ T cells and in advanced PDAC, with increased efficacy of radiation therapy [152].

A significant prognostic factor is the maturation state of cDCs. Mature cDCs in lung tertiary lymphoid structures are strongly associated with a Th1 and T cytotoxic immune signature, and long-term survival. The combination of mature cDCs and CD8^+^ T cell densities constitutes a powerful and independent prognostic factor of overall survival [153]. A block in maturation was found in metastasized breast cancer in cDCs localized in sentinel lymph nodes; this was associated with reduced co-localization of mature DC-CTL and expanded Treg cells [154]. In Glioblastoma Multiforme patients, cDC2 showed immature phenotype with reduced IL-12 production [155].

Immunohistochemistry analysis of infiltrated mature cDC subsets in primary colon adenocarcinoma showed accumulation of CD83^+^ cDCs mostly at the invasive margin, forming clusters with T lymphocytes [156]. In colorectal cancer, decreased levels of stroma mature DC-LAMP^+^ cells in association with reduced tumor infiltrating lymphocytes and increased immature/tolerogenic CD1a^+^ cDCs correlated with disease recurrence [157]. Consistent with this finding, infiltration of DC-LAMP^+^ cells in the TME of ovarian cancer patients [158] and cutaneous malignant melanoma were associated with better overall survival [159]. BDCA3^+^ DCs in melanoma patients correlated with improved overall survival and the formation of clusters with NK cells represents a prognostic tool for T cell-directed immunotherapy [43].

PDCs infiltration of TME is often linked to immune tolerance. Indeed, in epithelial ovarian cancer, pDCs drive immunosuppression mediated by the ICOS-L-dependent accumulation of Foxp3^+^ Treg cells, which localize in close proximity. The frequency of these two cell populations directly correlated with and was predictive of disease progression [160]. The accumulation of pDCs in ovarian cancer epithelium is an independent prognostic factor associated with early relapse [161]. PDCs can also induce lymphangiogenesis and neo-angiogenesis through the production of VEGF-C [162], TNF-α and IL-8 which are hallmarks of poor prognosis associated with multifocal intra-peritoneal dissemination [110]. In cutaneous melanoma, the presence of pDCs in draining lymph nodes is associated with poor prognosis [106].

Increased levels of pDCs in peripheral blood of Non-Small Cell Lung Cancer (NSCLC) patients and increased pDC infiltration in breast tumors and head and neck and oral squamous cell carcinomas correlated with more advanced tumor stages and poor prognosis [101,111,163,164,165,166]. 

MoDCs were detected in melanoma nodules [167] in lung and colon carcinoma both in mice and patients [49], and in ascites from ovarian and breast cancer patients with negative prognostic value [13].

## 6. Targeting DC Subsets to Improve Cancer Immunotherapy

The distinctive capacity of DCs to activate anti-tumor immune responses has been exploited in cancer immunotherapy [168,169,170,171,172]. As DCs are poorly present in circulation (about 1% of total leukocytes), most of the vaccination trials are based on moDCs, differentiated ex vivo from CD14^+^ monocytes or from CD34^+^ progenitors in the presence of growth factors (e.g., GM-CSF and IL-4) and maturation factors (such as TLR agonists or CD40L), loaded with different forms of tumor antigen preparation [172,173]. In the past two decades, several clinical studies on DC vaccination were conducted based on ex vivo generated DCs, such as the melanoma antigens-pulsed DC vaccines for metastatic melanoma patients [174,175], and the FDA-approved Sipuleucel-T for metastatic prostate cancer patients [176]. MoDC vaccines, tested in different cancer patients [177], demonstrated the ability of moDCs to cross-prime and activate T cells to produce antitumor cytokines in the presence of limited toxicity [178,179,180]. However, only a minority (5–15%) of metastatic patients showed objective clinical response notwithstanding a trend to survival benefit reported in most studies [181]. Of note, in vitro differentiated moDCs are distinct from the primary circulating DCs both at transcriptional and phenotypic levels [182] and function less efficiently in T cell priming and in migratory capacity [183,184]. Another reason for the apparent lack of success of moDC vaccines can be ascribed to enhanced tumor burden and immune suppression occurring in advanced malignancies.

From the pioneering studies by Ralph Steinman, who paved the way to new designed vaccines based on the principle of in vivo targeting DCs [185], recent advances in tumor immunology suggest to focus on autologous DCs and to exploit DC subset specificity [186]. Distinct primary DC subsets may be associated with improved survival in diverse types of cancer [25,32,42]. Considering that all DC subsets can generate an anti-tumor response, several clinical trials were conducted [187,188,189] and some are still ongoing (NCT02574377, NCT02692976, NCT02993315), using autologous primary cDC2s and pDCs [168]. Although cDC1s are the most promising DC subset, based on their ability to activate CD8^+^ T cells, their isolation from circulation still represents a major challenge that has limited clinical use [168]. The use of Flt3L-dependent mobilization can trigger an in vivo expansion of all DC subsets, including cDC1 [187,189]. Nevertheless, many attempts are under investigation in order to optimize cDC1-based vaccines for clinical applications. Recent work has highlighted the role of the Notch signaling pathway in driving cDC1 differentiation ex vivo [190,191]. Additionally, direct reprogramming of dermal fibroblasts into cDC1s might be a promising alternative strategy [192]. The efficacy of cDC1-based vaccine in preclinical settings was recently described and will pave the way to clinic [193].

Despite the revolution in cancer immunotherapy, the manipulation of DCs for cancer vaccines has not reached its full maturity. Optimization of molecular pathways to boost DC anti-tumor activity, such as maturation, priming, and trafficking, is still required. Recent evidence suggests that cell maturation in cDC1-based vaccines can be optimized using TLR3 (poly I:C) and TLR8 (R848) stimulation [194,195]. To improve T cell priming ability, several antigen loading approaches were explored. For example, moDCs pulsed with autologous oxidized whole tumor lysates led to prolonged patient survival in a personalized ovarian cancer clinical trial [196]. Alternatively, DCs, engineered with a chimeric receptor to increase selective uptake of tumor-derived, antigen-bearing extracellular vesicles, were tested in a preclinical model of DC vaccination and shown to produce expansion of specific T cells and effective anti-tumor response [197]. Antigen processing and cross-presentation can be improved through the regulation of proteolysis of internalized antigens as recently demonstrated by the genetic deletion of molecules involved in the vesicular trafficking machinery, such as SEC22B and YTHDF1 [30,198]. Another strategy that can be used to improve T cell priming is the silencing of cDC1-intrinsic immunosuppressive signals, by the use of small interfering RNA (siRNA) to delete PD-L1 and PD-L2 as already shown for moDCs [199]. The synergic use of DC vaccines and immune checkpoint blockade is one of the most promising therapeutic approach for cancer immunotherapy. The efficacy of anti-PD-1 and anti-TIM-3 checkpoint inhibitors is strictly correlated with indirect role of cDC1 that potentiate NK cell and CTL effector functions [43,94,200,201].

A very encouraging approach to improve the cross-presenting capacity of cDC1 is the direct targeting of CLEC9A, a molecule specifically expressed by cDC1, involved in the uptake of dead cells and cross-priming of antigens to CD8^+^ T cells [11,202]. In addition to antigen delivery, a CLEC9A antibody can also deliver maturation stimuli to cDC1, as shown by the intratumoral injection of a chimeric recombinant protein [203].

The ability of DCs to mount an anti-tumor immune response depends on their efficient tumor homing and migration to draining lymph nodes. CCR7 is the key player for DC migration to draining lymph nodes. The knowledge on how to induce CCR7 upregulation generated with moDCs, such as adenoviral transduction, immune adjuvant targeting micelles, and epigenetic regulation [204,205,206], should now be extended to cDC1-based vaccines [32]. Chemokines involved in the recruitment of cDC1 in tumors could be targeted to improve the clinical use of these cells. One valid example is the ability of adenovirally-induced CCL21-expressing DCs, injected intratumorally, to induce anti-tumor immunity in a phase I clinical trial in lung cancer [207]. The selective expression of XCR1 in both mice and humans cDC1 makes this molecule attractive for cDC1 targeting. XCR1 targeting was shown to be important in tumor antigen delivery to cDC1 and subsequent CD8^+^ T cell priming [208,209]. In addition, the intratumor inoculation of XCL1 increased cDC1 accumulation and improved tumor control in several preclinical models [34,42,210]. The usage of XCL1 variants engineered to enhance receptor stability and chemotactic activity was reported to further improve anti-tumor immune response [34] and could provide a rational for future translational developments.

## 7. Conclusions

DC subsets are diverse cell types that requires different transcription factors for their development, express specific membrane markers and possess specialized functions. The recognition of this complex scenario may allow to better understand their role in homeostatic and pathological conditions and to develop successful therapeutic approaches. DCs exert a major role in the control of anti-tumor immune response. This function is accomplished through efficient antigen processing and presentation and cytokine production. However, tumor-derived molecules may decrease their anti-tumor potential and turn DCs in cells with tumor-promoting activity. Based on their specialized ability to cross-present tumor antigens to CD8 T cells and to their homing characteristics, cDC1s are emerging as the DC subset endowed with the highest potential to activate an anti-tumor response, both in mouse and human settings. At present, the most common approach in DC-based cancer therapy rely on the use of moDCs. These cells showed limited efficacy in terms of cross-presentation and migration to lymph nodes. In addition, moDCs can turned out to favor an immunosuppressive context in TME [168]. Primary DCs are considered a potent alternative to moDCs, especially cDC1s. However, successful DC-targeted immunotherapy might require co-administration with other drugs, in a multiphase approach, such as in association with chemotherapy, radiotherapy, or checkpoint inhibitors [173]. Chemotherapy can enhance anti-tumor functions through multiple mechanisms, including the release of tumor associated antigens and the depletion of the tumor suppressive milieu [72,73]. Additionally, radiotherapy can enhance response to immunotherapy and can promote immune-mediated tumor clearance by a mechanism apparently associated with radiation-induced cytosolic DNA, which stimulates the secretion of IFN-β by cancer cells. IFN-β promotes activation and recruitment of cDC1, responsible for CD8^+^ T cell priming and systemic tumor rejection (abscopal effect) in the context of immune checkpoint inhibition [211].

Tumor-induced immunosuppression is one of the most important challenge for DC vaccination, and the association with immune checkpoint inhibition is under evaluation in clinical trials [173]. A better understanding of the specialized immune functions of DC subsets will improve our understanding of their role in pathology and will provide the rationale for the design of new therapeutic strategies.

## Abbreviations

DCs	Dendritic Cells
PAMPs	Pathogen-Associated Molecular Patterns
DAMPs	Damage-Associated Molecular Patterns
cDCs	Conventional DCs
pDCs	Plasmacytoid DCs
moDCs	Monocyte-derived DCs
IRF8	Interferon Regulatory Factor 8
ID2	Inhibitor DNA-binding 2 protein HLH
BATF3	Basic leucine zipper ATF-like Transcription Factor 3
IRF4	Interferon Regulatory Factor 4
ZEB	Zinc ginger E-box-binding homeobox1
KLF4	Kruppel-Like Factor 4
DNGR-1	Dendritic cell Natural killer lectin Group Receptor-1
CLEC9A	C-type lectin domain family 9 member A
MHC	Major Histocompatibility Complex
BDCA	Blood Dendritic Cell Antigens
IFN	Interferon
TME	Tumor Microenvironment
CTL	Cytotoxic T Lymphocytes
SNARE	SNAP Receptor (SNAP= Soluble NSF Attachment Protein)
IL-12	Interleukin 12
NK	Natural Killer
cGAS	Cyclic GMP-AMP Synthase
STING	Stimulator of Interferon Genes
Flt3L	FMS-like tyrosine kinase 3 Ligand
IFNAR1	Interferon-alpha receptor alpha chain
TRAIL	TNF-related apoptosis-inducing ligand
TLR	Toll Like Receptor
PGE2	Prostaglandin E2
VEGF	Vascular Endothelial Growth Factor
CSF-1	Colony Stimulating Factor 1
COX	Cyclooxygenase
IDO	Indoleamine 2,3 dioxygenase
HMGB1	High Mobility Group Box 1
TIM3	T-cell immunoglobulin and mucin-domain containing-3
HLA-DR	Human Leukocyte Antigen-DR isotype
PD-L	Programmed Death-Ligand
PD-1	Programmed cell Death protein 1
MGL2	Macrophage Galactose N-acetyl-galactosamine specific Lectin 2 isoform
Treg	Regulatory T cell
XBP1	X-Box binding protein 1
EV	Extracellular vesicle
STAT3	Signal Transducer and Activator of Transcription 3
ICOSL	Inducible T cell co-stimulator ligand
TGF-β	Transforming Growth Factor beta
TNF-α	Tumor Necrosis Factor alpha
ROS	Reactive Oxygen Species
CRAMP	Cathelicidin-Related AntiMicrobial Peptide
FPR1	Formyl Peptide Receptor 1
CMKLR1	Chemokine Like Receptor 1
ATP	Adenosine triphosphate
LBC	Luminal Breast Cancer
TNBC	Triple Negative Breast Cancer
PDAC	Pancreatic Ductal AdenoCarcinoma
LAMP	Lysosomal-Associated Membrane Protein
NSCLC	Non-Small Cell Lung Cancer
YTHDF1	YTH domain-containing family protein
siRNA	Small interfering RNA

## References

[1] Banchereau J., Briere F., Caux C., Davoust J., Lebecque S., Liu Y.J., Pulendran B., Palucka K. (2000). Immunobiology of dendritic cells. Annu. Rev. Immunol..

[2] Steinman R.M. (2012). Decisions about dendritic cells: Past, present, and future. Annu. Rev. Immunol..

[3] Schlitzer A., McGovern N., Ginhoux F. (2015). Dendritic cells and monocyte-derived cells: Two complementary and integrated functional systems. Semin. Cell Dev. Biol..

[4] Merad M., Sathe P., Helft J., Miller J., Mortha A. (2013). The dendritic cell lineage: Ontogeny and function of dendritic cells and their subsets in the steady state and the inflamed setting. Annu. Rev. Immunol..

[5] Mildner A., Jung S. (2014). Development and function of dendritic cell subsets. Immunity.

[6] Segura E. (2016). Review of Mouse and Human Dendritic Cell Subsets. Methods Mol. Biol..

[7] Collin M., Bigley V. (2018). Human dendritic cell subsets: an update. Immunology.

[8] Guilliams M., Dutertre C.A., Scott C.L., McGovern N., Sichien D., Chakarov S., Van Gassen S., Chen J., Poidinger M., De Prijck S. (2016). Unsupervised High-Dimensional Analysis Aligns Dendritic Cells across Tissues and Species. Immunity.

[9] Crozat K., Tamoutounour S., Vu Manh T.P., Fossum E., Luche H., Ardouin L., Guilliams M., Azukizawa H., Bogen B., Malissen B. (2011). Cutting edge: Expression of XCR1 defines mouse lymphoid-tissue resident and migratory dendritic cells of the CD8alpha+ type. J. Immunol..

[10] Poulin L.F., Reyal Y., Uronen-Hansson H., Schraml B.U., Sancho D., Murphy K.M., Hakansson U.K., Moita L.F., Agace W.W., Bonnet D. (2012). DNGR-1 is a specific and universal marker of mouse and human Batf3-dependent dendritic cells in lymphoid and nonlymphoid tissues. Blood.

[11] Cueto F.J., Del Fresno C., Sancho D. (2019). DNGR-1 A Dendritic Cell-Specific Sensor of Tissue Damage That Dually Modulates Immunity and Inflammation. Front. Immunol..

[12] Facchetti F., Vermi W., Mason D., Colonna M. (2003). The plasmacytoid monocyte/interferon producing cells. Virchows Arch..

[13] Segura E., Touzot M., Bohineust A., Cappuccio A., Chiocchia G., Hosmalin A., Dalod M., Soumelis V., Amigorena S. (2013). Human inflammatory dendritic cells induce Th17 cell differentiation. Immunity.

[14] Villani A.C., Satija R., Reynolds G., Sarkizova S., Shekhar K., Fletcher J., Griesbeck M., Butler A., Zheng S., Lazo S. (2017). Single-cell RNA-seq reveals new types of human blood dendritic cells, monocytes, and progenitors. Science.

[15] Tiberio L., Del Prete A., Schioppa T., Sozio F., Bosisio D., Sozzani S. (2018). Chemokine and chemotactic signals in dendritic cell migration. Cell Mol. Immunol..

[16] Sozzani S., Vermi W., Del Prete A., Facchetti F. (2010). Trafficking properties of plasmacytoid dendritic cells in health and disease. Trends Immunol..

[17] Bosisio D., Ronca R., Salvi V., Presta M., Sozzani S. (2018). Dendritic cells in inflammatory angiogenesis and lymphangiogenesis. Curr. Opin. Immunol..

[18] Sozzani S., Del Prete A., Bosisio D. (2017). Dendritic cell recruitment and activation in autoimmunity. J. Autoimmun.

[19] Gardner A., Ruffell B. (2016). Dendritic Cells and Cancer Immunity. Trends Immunol..

[20] Faget J., Bendriss-Vermare N., Gobert M., Durand I., Olive D., Biota C., Bachelot T., Treilleux I., Goddard-Leon S., Lavergne E. (2012). ICOS-ligand expression on plasmacytoid dendritic cells supports breast cancer progression by promoting the accumulation of immunosuppressive CD4+ T cells. Cancer Res..

[21] Vu Manh T.P., Bertho N., Hosmalin A., Schwartz-Cornil I., Dalod M. (2015). Investigating Evolutionary Conservation of Dendritic Cell Subset Identity and Functions. Front. Immunol..

[22] Cancel J.C., Crozat K., Dalod M., Mattiuz R. (2019). Are Conventional Type 1 Dendritic Cells Critical for Protective Antitumor Immunity and How?. Front. Immunol..

[23] Bottcher J.P., Reis E.S.C. (2018). The Role of Type 1 Conventional Dendritic Cells in Cancer Immunity. Trends Cancer.

[24] Hildner K., Edelson B.T., Purtha W.E., Diamond M., Matsushita H., Kohyama M., Calderon B., Schraml B.U., Unanue E.R., Diamond M.S. (2008). Batf3 deficiency reveals a critical role for CD8alpha+ dendritic cells in cytotoxic T cell immunity. Science.

[25] Broz M.L., Binnewies M., Boldajipour B., Nelson A.E., Pollack J.L., Erle D.J., Barczak A., Rosenblum M.D., Daud A., Barber D.L. (2014). Dissecting the tumor myeloid compartment reveals rare activating antigen-presenting cells critical for T cell immunity. Cancer Cell.

[26] Sanchez-Paulete A.R., Cueto F.J., Martinez-Lopez M., Labiano S., Morales-Kastresana A., Rodriguez-Ruiz M.E., Jure-Kunkel M., Azpilikueta A., Aznar M.A., Quetglas J.I. (2016). Cancer Immunotherapy with Immunomodulatory Anti-CD137 and Anti-PD-1 Monoclonal Antibodies Requires BATF3-Dependent Dendritic Cells. Cancer Discov..

[27] Salmon H., Idoyaga J., Rahman A., Leboeuf M., Remark R., Jordan S., Casanova-Acebes M., Khudoynazarova M., Agudo J., Tung N. (2016). Expansion and Activation of CD103(+) Dendritic Cell Progenitors at the Tumor Site Enhances Tumor Responses to Therapeutic PD-L1 and BRAF Inhibition. Immunity.

[28] Spranger S., Dai D., Horton B., Gajewski T.F. (2017). Tumor-Residing Batf3 Dendritic Cells Are Required for Effector T Cell Trafficking and Adoptive T Cell Therapy. Cancer Cell.

[29] Theisen D.J., Davidson J.T.T., Briseno C.G., Gargaro M., Lauron E.J., Wang Q., Desai P., Durai V., Bagadia P., Brickner J.R. (2018). WDFY4 is required for cross-presentation in response to viral and tumor antigens. Science.

[30] Alloatti A., Rookhuizen D.C., Joannas L., Carpier J.M., Iborra S., Magalhaes J.G., Yatim N., Kozik P., Sancho D., Albert M.L. (2017). Critical role for Sec22b-dependent antigen cross-presentation in antitumor immunity. J. Exp. Med..

[31] Theisen D.J., Ferris S.T., Briseno C.G., Kretzer N., Iwata A., Murphy K.M., Murphy T.L. (2019). Batf3-Dependent Genes Control Tumor Rejection Induced by Dendritic Cells Independently of Cross-Presentation. Cancer Immunol. Res..

[32] Roberts E.W., Broz M.L., Binnewies M., Headley M.B., Nelson A.E., Wolf D.M., Kaisho T., Bogunovic D., Bhardwaj N., Krummel M.F. (2016). Critical Role for CD103(+)/CD141(+) Dendritic Cells Bearing CCR7 for Tumor Antigen Trafficking and Priming of T Cell Immunity in Melanoma. Cancer Cell.

[33] Kroczek R.A., Henn V. (2012). The Role of XCR1 and its Ligand XCL1 in Antigen Cross-Presentation by Murine and Human Dendritic Cells. Front. Immunol..

[34] Matsuo K., Kitahata K., Kawabata F., Kamei M., Hara Y., Takamura S., Oiso N., Kawada A., Yoshie O., Nakayama T. (2018). A Highly Active Form of XCL1/Lymphotactin Functions as an Effective Adjuvant to Recruit Cross-Presenting Dendritic Cells for Induction of Effector and Memory CD8(+) T Cells. Front. Immunol..

[35] Mikucki M.E., Fisher D.T., Matsuzaki J., Skitzki J.J., Gaulin N.B., Muhitch J.B., Ku A.W., Frelinger J.G., Odunsi K., Gajewski T.F. (2015). Non-redundant requirement for CXCR3 signalling during tumoricidal T-cell trafficking across tumour vascular checkpoints. Nat. Commun..

[36] Kastenmuller W., Brandes M., Wang Z., Herz J., Egen J.G., Germain R.N. (2013). Peripheral prepositioning and local CXCL9 chemokine-mediated guidance orchestrate rapid memory CD8+ T cell responses in the lymph node. Immunity.

[37] Enamorado M., Iborra S., Priego E., Cueto F.J., Quintana J.A., Martinez-Cano S., Mejias-Perez E., Esteban M., Melero I., Hidalgo A. (2017). Enhanced anti-tumour immunity requires the interplay between resident and circulating memory CD8(+) T cells. Nat. Commun..

[38] Mittal D., Vijayan D., Putz E.M., Aguilera A.R., Markey K.A., Straube J., Kazakoff S., Nutt S.L., Takeda K., Hill G.R. (2017). Interleukin-12 from CD103(+) Batf3-Dependent Dendritic Cells Required for NK-Cell Suppression of Metastasis. Cancer Immunol. Res..

[39] Ruffell B., Chang-Strachan D., Chan V., Rosenbusch A., Ho C.M., Pryer N., Daniel D., Hwang E.S., Rugo H.S., Coussens L.M. (2014). Macrophage IL-10 blocks CD8+ T cell-dependent responses to chemotherapy by suppressing IL-12 expression in intratumoral dendritic cells. Cancer Cell.

[40] Greyer M., Whitney P.G., Stock A.T., Davey G.M., Tebartz C., Bachem A., Mintern J.D., Strugnell R.A., Turner S.J., Gebhardt T. (2016). T Cell Help Amplifies Innate Signals in CD8(+) DCs for Optimal CD8(+) T Cell Priming. Cell Rep..

[41] Deauvieau F., Ollion V., Doffin A.C., Achard C., Fonteneau J.F., Verronese E., Durand I., Ghittoni R., Marvel J., Dezutter-Dambuyant C. (2015). Human natural killer cells promote cross-presentation of tumor cell-derived antigens by dendritic cells. Int. J. Cancer.

[42] Bottcher J.P., Bonavita E., Chakravarty P., Blees H., Cabeza-Cabrerizo M., Sammicheli S., Rogers N.C., Sahai E., Zelenay S., Reis e Sousa C. (2018). NK Cells Stimulate Recruitment of cDC1 into the Tumor Microenvironment Promoting Cancer Immune Control. Cell.

[43] Barry K.C., Hsu J., Broz M.L., Cueto F.J., Binnewies M., Combes A.J., Nelson A.E., Loo K., Kumar R., Rosenblum M.D. (2018). A natural killer-dendritic cell axis defines checkpoint therapy-responsive tumor microenvironments. Nat. Med..

[44] Hor J.L., Whitney P.G., Zaid A., Brooks A.G., Heath W.R., Mueller S.N. (2015). Spatiotemporally Distinct Interactions with Dendritic Cell Subsets Facilitates CD4+ and CD8+ T Cell Activation to Localized Viral Infection. Immunity.

[45] Brewitz A., Eickhoff S., Dahling S., Quast T., Bedoui S., Kroczek R.A., Kurts C., Garbi N., Barchet W., Iannacone M. (2017). CD8(+) T Cells Orchestrate pDC-XCR1(+) Dendritic Cell Spatial and Functional Cooperativity to Optimize Priming. Immunity.

[46] Diamond M.S., Kinder M., Matsushita H., Mashayekhi M., Dunn G.P., Archambault J.M., Lee H., Arthur C.D., White J.M., Kalinke U. (2011). Type I interferon is selectively required by dendritic cells for immune rejection of tumors. J. Exp. Med..

[47] Fuertes M.B., Kacha A.K., Kline J., Woo S.R., Kranz D.M., Murphy K.M., Gajewski T.F. (2011). Host type I IFN signals are required for antitumor CD8+ T cell responses through CD8{alpha}+ dendritic cells. J. Exp. Med..

[48] Marcus A., Mao A.J., Lensink-Vasan M., Wang L., Vance R.E., Raulet D.H. (2018). Tumor-Derived cGAMP Triggers a STING-Mediated Interferon Response in Non-tumor Cells to Activate the NK Cell Response. Immunity.

[49] Laoui D., Keirsse J., Morias Y., Van Overmeire E., Geeraerts X., Elkrim Y., Kiss M., Bolli E., Lahmar Q., Sichien D. (2016). The tumour microenvironment harbours ontogenically distinct dendritic cell populations with opposing effects on tumour immunity. Nat. Commun..

[50] Qin Z., Blankenstein T. (2000). CD4+ T cell--mediated tumor rejection involves inhibition of angiogenesis that is dependent on IFN gamma receptor expression by nonhematopoietic cells. Immunity.

[51] Quezada S.A., Simpson T.R., Peggs K.S., Merghoub T., Vider J., Fan X., Blasberg R., Yagita H., Muranski P., Antony P.A. (2010). Tumor-reactive CD4(+) T cells develop cytotoxic activity and eradicate large established melanoma after transfer into lymphopenic hosts. J. Exp. Med..

[52] Kim H.J., Cantor H. (2014). CD4 T-cell subsets and tumor immunity: the helpful and the not-so-helpful. Cancer Immunol. Res..

[53] Kennedy R., Celis E. (2008). Multiple roles for CD4+ T cells in anti-tumor immune responses. Immunol. Rev..

[54] Borst J., Ahrends T., Babala N., Melief C.J.M., Kastenmuller W. (2018). CD4(+) T cell help in cancer immunology and immunotherapy. Nat. Rev. Immunol..

[55] Amigorena S. (2015). Helping the Help for CD8+ T Cell Responses. Cell.

[56] Janssen E.M., Lemmens E.E., Wolfe T., Christen U., von Herrath M.G., Schoenberger S.P. (2003). CD4+ T cells are required for secondary expansion and memory in CD8+ T lymphocytes. Nature.

[57] Binnewies M., Mujal A.M., Pollack J.L., Combes A.J., Hardison E.A., Barry K.C., Tsui J., Ruhland M.K., Kersten K., Abushawish M.A. (2019). Unleashing Type-2 Dendritic Cells to Drive Protective Antitumor CD4(+) T Cell Immunity. Cell.

[58] Leal Rojas I.M., Mok W.H., Pearson F.E., Minoda Y., Kenna T.J., Barnard R.T., Radford K.J. (2017). Human Blood CD1c(+) Dendritic Cells Promote Th1 and Th17 Effector Function in Memory CD4(+) T Cells. Front. Immunol..

[59] Anandasabapathy N., Breton G., Hurley A., Caskey M., Trumpfheller C., Sarma P., Pring J., Pack M., Buckley N., Matei I. (2015). Efficacy and safety of CDX-301, recombinant human Flt3L, at expanding dendritic cells and hematopoietic stem cells in healthy human volunteers. Bone Marrow Transplant..

[60] Pinzon-Charry A., Ho C.S., Maxwell T., McGuckin M.A., Schmidt C., Furnival C., Pyke C.M., Lopez J.A. (2007). Numerical and functional defects of blood dendritic cells in early- and late-stage breast cancer. Br. J. Cancer.

[61] Lavin Y., Kobayashi S., Leader A., Amir E.D., Elefant N., Bigenwald C., Remark R., Sweeney R., Becker C.D., Levine J.H. (2017). Innate Immune Landscape in Early Lung Adenocarcinoma by Paired Single-Cell Analyses. Cell.

[62] Michea P., Noel F., Zakine E., Czerwinska U., Sirven P., Abouzid O., Goudot C., Scholer-Dahirel A., Vincent-Salomon A., Reyal F. (2018). Adjustment of dendritic cells to the breast-cancer microenvironment is subset specific. Nat. Immunol..

[63] Zilionis R., Engblom C., Pfirschke C., Savova V., Zemmour D., Saatcioglu H.D., Krishnan I., Maroni G., Meyerovitz C.V., Kerwin C.M. (2019). Single-Cell Transcriptomics of Human and Mouse Lung Cancers Reveals Conserved Myeloid Populations across Individuals and Species. Immunity.

[64] Koucky V., Boucek J., Fialova A. (2019). Immunology of Plasmacytoid Dendritic Cells in Solid Tumors: A Brief Review. Cancers (Basel).

[65] Riboldi E., Daniele R., Cassatella M.A., Sozzani S., Bosisio D. (2009). Engagement of BDCA-2 blocks TRAIL-mediated cytotoxic activity of plasmacytoid dendritic cells. Immunobiology.

[66] Salvi V., Vermi W., Cavani A., Lonardi S., Carbone T., Facchetti F., Bosisio D., Sozzani S. (2017). IL-21 May Promote Granzyme B-Dependent NK/Plasmacytoid Dendritic Cell Functional Interaction in Cutaneous Lupus Erythematosus. J. Investig. Dermatol..

[67] Drobits B., Holcmann M., Amberg N., Swiecki M., Grundtner R., Hammer M., Colonna M., Sibilia M. (2012). Imiquimod clears tumors in mice independent of adaptive immunity by converting pDCs into tumor-killing effector cells. J. Clin. Investig..

[68] Wu J., Li S., Yang Y., Zhu S., Zhang M., Qiao Y., Liu Y.J., Chen J. (2017). TLR-activated plasmacytoid dendritic cells inhibit breast cancer cell growth in vitro and in vivo. Oncotarget.

[69] Liu C., Lou Y., Lizee G., Qin H., Liu S., Rabinovich B., Kim G.J., Wang Y.H., Ye Y., Sikora A.G. (2008). Plasmacytoid dendritic cells induce NK cell-dependent, tumor antigen-specific T cell cross-priming and tumor regression in mice. J. Clin. Investig..

[70] Poropatich K.O., Dominguez D., Chan W.C., Andrade J., Zha Y., Wray B.D., Miska J., Qin L., Cole L.E., Coates S. (2020). OX40+ plasmacytoid dendritic cells in the tumor microenvironment promote antitumor immunity. J. Clin. Investig..

[71] Kuhn S., Yang J., Ronchese F. (2015). Monocyte-Derived Dendritic Cells Are Essential for CD8(+) T Cell Activation and Antitumor Responses After Local Immunotherapy. Front. Immunol..

[72] Ma Y., Adjemian S., Mattarollo S.R., Yamazaki T., Aymeric L., Yang H., Portela Catani J.P., Hannani D., Duret H., Steegh K. (2013). Anticancer chemotherapy-induced intratumoral recruitment and differentiation of antigen-presenting cells. Immunity.

[73] Pfirschke C., Engblom C., Rickelt S., Cortez-Retamozo V., Garris C., Pucci F., Yamazaki T., Poirier-Colame V., Newton A., Redouane Y. (2016). Immunogenic Chemotherapy Sensitizes Tumors to Checkpoint Blockade Therapy. Immunity.

[74] Diao J., Gu H., Tang M., Zhao J., Cattral M.S. (2018). Tumor Dendritic Cells (DCs) Derived from Precursors of Conventional DCs Are Dispensable for Intratumor CTL Responses. J. Immunol..

[75] DeVito N.C., Plebanek M.P., Theivanthiran B., Hanks B.A. (2019). Role of Tumor-Mediated Dendritic Cell Tolerization in Immune Evasion. Front. Immunol..

[76] Gabrilovich D.I., Chen H.L., Girgis K.R., Cunningham H.T., Meny G.M., Nadaf S., Kavanaugh D., Carbone D.P. (1996). Production of vascular endothelial growth factor by human tumors inhibits the functional maturation of dendritic cells. Nat. Med..

[77] Kaplan R.N., Riba R.D., Zacharoulis S., Bramley A.H., Vincent L., Costa C., MacDonald D.D., Jin D.K., Shido K., Kerns S.A. (2005). VEGFR1-positive haematopoietic bone marrow progenitors initiate the pre-metastatic niche. Nature.

[78] Conejo-Garcia J.R., Benencia F., Courreges M.C., Kang E., Mohamed-Hadley A., Buckanovich R.J., Holtz D.O., Jenkins A., Na H., Zhang L. (2004). Tumor-infiltrating dendritic cell precursors recruited by a beta-defensin contribute to vasculogenesis under the influence of Vegf-A. Nat. Med..

[79] Tang M., Diao J., Gu H., Khatri I., Zhao J., Cattral M.S. (2015). Toll-like Receptor 2 Activation Promotes Tumor Dendritic Cell Dysfunction by Regulating IL-6 and IL-10 Receptor Signaling. Cell Rep..

[80] Vulcano M., Albanesi C., Stoppacciaro A., Bagnati R., D’Amico G., Struyf S., Transidico P., Bonecchi R., Del Prete A., Allavena P. (2001). Dendritic cells as a major source of macrophage-derived chemokine/CCL22 in vitro and in vivo. Eur. J. Immunol..

[81] D’Ambrosio D., Iellem A., Bonecchi R., Mazzeo D., Sozzani S., Mantovani A., Sinigaglia F. (1998). Selective up-regulation of chemokine receptors CCR4 and CCR8 upon activation of polarized human type 2 Th cells. J. Immunol..

[82] Bauer C.A., Kim E.Y., Marangoni F., Carrizosa E., Claudio N.M., Mempel T.R. (2014). Dynamic Treg interactions with intratumoral APCs promote local CTL dysfunction. J. Clin. Investig..

[83] Rohrle N., Knott M.M.L., Anz D. (2020). CCL22 Signaling in the Tumor Environment. Adv. Exp. Med. Biol..

[84] Zelenay S., van der Veen A.G., Bottcher J.P., Snelgrove K.J., Rogers N., Acton S.E., Chakravarty P., Girotti M.R., Marais R., Quezada S.A. (2015). Cyclooxygenase-Dependent Tumor Growth through Evasion of Immunity. Cell.

[85] Munn D.H., Mellor A.L. (2016). IDO in the Tumor Microenvironment: Inflammation, Counter-Regulation, and Tolerance. Trends Immunol..

[86] Caronni N., Simoncello F., Stafetta F., Guarnaccia C., Ruiz-Moreno J.S., Opitz B., Galli T., Proux-Gillardeaux V., Benvenuti F. (2018). Downregulation of Membrane Trafficking Proteins and Lactate Conditioning Determine Loss of Dendritic Cell Function in Lung Cancer. Cancer Res..

[87] Herber D.L., Cao W., Nefedova Y., Novitskiy S.V., Nagaraj S., Tyurin V.A., Corzo A., Cho H.I., Celis E., Lennox B. (2010). Lipid accumulation and dendritic cell dysfunction in cancer. Nat. Med..

[88] Ramakrishnan R., Tyurin V.A., Veglia F., Condamine T., Amoscato A., Mohammadyani D., Johnson J.J., Zhang L.M., Klein-Seetharaman J., Celis E. (2014). Oxidized lipids block antigen cross-presentation by dendritic cells in cancer. J. Immunol..

[89] Cubillos-Ruiz J.R., Silberman P.C., Rutkowski M.R., Chopra S., Perales-Puchalt A., Song M., Zhang S., Bettigole S.E., Gupta D., Holcomb K. (2015). ER Stress Sensor XBP1 Controls Anti-tumor Immunity by Disrupting Dendritic Cell Homeostasis. Cell.

[90] Kudo-Saito C., Shirako H., Ohike M., Tsukamoto N., Kawakami Y. (2013). CCL2 is critical for immunosuppression to promote cancer metastasis. Clin. Exp. Metastasis.

[91] Lim T.S., Chew V., Sieow J.L., Goh S., Yeong J.P., Soon A.L., Ricciardi-Castagnoli P. (2016). PD-1 expression on dendritic cells suppresses CD8(+) T cell function and antitumor immunity. Oncoimmunology.

[92] Krempski J., Karyampudi L., Behrens M.D., Erskine C.L., Hartmann L., Dong H., Goode E.L., Kalli K.R., Knutson K.L. (2011). Tumor-infiltrating programmed death receptor-1+ dendritic cells mediate immune suppression in ovarian cancer. J. Immunol..

[93] Francisco L.M., Salinas V.H., Brown K.E., Vanguri V.K., Freeman G.J., Kuchroo V.K., Sharpe A.H. (2009). PD-L1 regulates the development, maintenance, and function of induced regulatory T cells. J. Exp. Med..

[94] De Mingo Pulido A., Gardner A., Hiebler S., Soliman H., Rugo H.S., Krummel M.F., Coussens L.M., Ruffell B. (2018). TIM-3 Regulates CD103(+) Dendritic Cell Function and Response to Chemotherapy in Breast Cancer. Cancer Cell.

[95] Chiba S., Baghdadi M., Akiba H., Yoshiyama H., Kinoshita I., Dosaka-Akita H., Fujioka Y., Ohba Y., Gorman J.V., Colgan J.D. (2012). Tumor-infiltrating DCs suppress nucleic acid-mediated innate immune responses through interactions between the receptor TIM-3 and the alarmin HMGB1. Nat. Immunol..

[96] Kenkel J.A., Tseng W.W., Davidson M.G., Tolentino L.L., Choi O., Bhattacharya N., Seeley E.S., Winer D.A., Reticker-Flynn N.E., Engleman E.G. (2017). An Immunosuppressive Dendritic Cell Subset Accumulates at Secondary Sites and Promotes Metastasis in Pancreatic Cancer. Cancer Res..

[97] Kalluri R. (2016). The biology and function of exosomes in cancer. J. Clin. Investig..

[98] Czystowska-Kuzmicz M., Sosnowska A., Nowis D., Ramji K., Szajnik M., Chlebowska-Tuz J., Wolinska E., Gaj P., Grazul M., Pilch Z. (2019). Small extracellular vesicles containing arginase-1 suppress T-cell responses and promote tumor growth in ovarian carcinoma. Nat. Commun..

[99] Shen Y., Guo D., Weng L., Wang S., Ma Z., Yang Y., Wang P., Wang J., Cai Z. (2017). Tumor-derived exosomes educate dendritic cells to promote tumor metastasis via HSP72/HSP105-TLR2/TLR4 pathway. Oncoimmunology.

[100] Ding G., Zhou L., Qian Y., Fu M., Chen J., Chen J., Xiang J., Wu Z., Jiang G., Cao L. (2015). Pancreatic cancer-derived exosomes transfer miRNAs to dendritic cells and inhibit RFXAP expression via miR-212-3p. Oncotarget.

[101] Hartmann E., Wollenberg B., Rothenfusser S., Wagner M., Wellisch D., Mack B., Giese T., Gires O., Endres S., Hartmann G. (2003). Identification and functional analysis of tumor-infiltrating plasmacytoid dendritic cells in head and neck cancer. Cancer Res..

[102] Demoulin S., Herfs M., Somja J., Roncarati P., Delvenne P., Hubert P. (2015). HMGB1 secretion during cervical carcinogenesis promotes the acquisition of a tolerogenic functionality by plasmacytoid dendritic cells. Int. J. Cancer.

[103] Ito T., Yang M., Wang Y.H., Lande R., Gregorio J., Perng O.A., Qin X.F., Liu Y.J., Gilliet M. (2007). Plasmacytoid dendritic cells prime IL-10-producing T regulatory cells by inducible costimulator ligand. J. Exp. Med..

[104] Wei S., Kryczek I., Zou L., Daniel B., Cheng P., Mottram P., Curiel T., Lange A., Zou W. (2005). Plasmacytoid dendritic cells induce CD8+ regulatory T cells in human ovarian carcinoma. Cancer Res..

[105] Vermi W., Soncini M., Melocchi L., Sozzani S., Facchetti F. (2011). Plasmacytoid dendritic cells and cancer. J. Leukoc Biol..

[106] Aspord C., Leccia M.T., Charles J., Plumas J. (2013). Plasmacytoid dendritic cells support melanoma progression by promoting Th2 and regulatory immunity through OX40L and ICOSL. Cancer Immunol. Res..

[107] Terra M., Oberkampf M., Fayolle C., Rosenbaum P., Guillerey C., Dadaglio G., Leclerc C. (2018). Tumor-Derived TGFbeta Alters the Ability of Plasmacytoid Dendritic Cells to Respond to Innate Immune Signaling. Cancer Res..

[108] Sharma M.D., Baban B., Chandler P., Hou D.Y., Singh N., Yagita H., Azuma M., Blazar B.R., Mellor A.L., Munn D.H. (2007). Plasmacytoid dendritic cells from mouse tumor-draining lymph nodes directly activate mature Tregs via indoleamine 2,3-dioxygenase. J. Clin. Investig..

[109] Chevolet I., Speeckaert R., Schreuer M., Neyns B., Krysko O., Bachert C., Hennart B., Allorge D., van Geel N., Van Gele M. (2015). Characterization of the in vivo immune network of IDO, tryptophan metabolism, PD-L1, and CTLA-4 in circulating immune cells in melanoma. Oncoimmunology.

[110] Curiel T.J., Cheng P., Mottram P., Alvarez X., Moons L., Evdemon-Hogan M., Wei S., Zou L., Kryczek I., Hoyle G. (2004). Dendritic cell subsets differentially regulate angiogenesis in human ovarian cancer. Cancer Res..

[111] Sorrentino R., Terlizzi M., Di Crescenzo V.G., Popolo A., Pecoraro M., Perillo G., Galderisi A., Pinto A. (2015). Human lung cancer-derived immunosuppressive plasmacytoid dendritic cells release IL-1alpha in an AIM2 inflammasome-dependent manner. Am. J. Pathol..

[112] Jahrsdorfer B., Vollmer A., Blackwell S.E., Maier J., Sontheimer K., Beyer T., Mandel B., Lunov O., Tron K., Nienhaus G.U. (2010). Granzyme B produced by human plasmacytoid dendritic cells suppresses T-cell expansion. Blood.

[113] Tesone A.J., Rutkowski M.R., Brencicova E., Svoronos N., Perales-Puchalt A., Stephen T.L., Allegrezza M.J., Payne K.K., Nguyen J.M., Wickramasinghe J. (2016). Satb1 Overexpression Drives Tumor-Promoting Activities in Cancer-Associated Dendritic Cells. Cell Rep..

[114] Toniolo P.A., Liu S., Yeh J.E., Ye D.Q., Barbuto J.A., Frank D.A. (2016). Deregulation of SOCS5 suppresses dendritic cell function in chronic lymphocytic leukemia. Oncotarget.

[115] Brown S., Hutchinson C.V., Aspinall-O’Dea M., Whetton A.D., Johnson S.M., Rees-Unwin K., Burthem J. (2014). Monocyte-derived dendritic cells from chronic myeloid leukaemia have abnormal maturation and cytoskeletal function that is associated with defective localisation and signalling by normal ABL1 protein. Eur. J. Haematol..

[116] Nathan C., Cunningham-Bussel A. (2013). Beyond oxidative stress: an immunologist’s guide to reactive oxygen species. Nat. Rev. Immunol..

[117] Reczek C.R., Chandel N.S. (2018). ROS Promotes Cancer Cell Survival through Calcium Signaling. Cancer Cell.

[118] Sozzani S. (2005). Dendritic cell trafficking: more than just chemokines. Cytokine Growth Factor Rev..

[119] Nagarsheth N., Wicha M.S., Zou W. (2017). Chemokines in the cancer microenvironment and their relevance in cancer immunotherapy. Nat. Rev. Immunol..

[120] Diao J., Zhao J., Winter E., Cattral M.S. (2010). Recruitment and differentiation of conventional dendritic cell precursors in tumors. J. Immunol..

[121] Tan M.C., Goedegebuure P.S., Belt B.A., Flaherty B., Sankpal N., Gillanders W.E., Eberlein T.J., Hsieh C.S., Linehan D.C. (2009). Disruption of CCR5-dependent homing of regulatory T cells inhibits tumor growth in a murine model of pancreatic cancer. J. Immunol..

[122] Halama N., Zoernig I., Berthel A., Kahlert C., Klupp F., Suarez-Carmona M., Suetterlin T., Brand K., Krauss J., Lasitschka F. (2016). Tumoral Immune Cell Exploitation in Colorectal Cancer Metastases Can Be Targeted Effectively by Anti-CCR5 Therapy in Cancer Patients. Cancer Cell.

[123] Bachem A., Hartung E., Guttler S., Mora A., Zhou X., Hegemann A., Plantinga M., Mazzini E., Stoitzner P., Gurka S. (2012). Expression of XCR1 Characterizes the Batf3-Dependent Lineage of Dendritic Cells Capable of Antigen Cross-Presentation. Front. Immunol..

[124] Dorner B.G., Dorner M.B., Zhou X., Opitz C., Mora A., Guttler S., Hutloff A., Mages H.W., Ranke K., Schaefer M. (2009). Selective expression of the chemokine receptor XCR1 on cross-presenting dendritic cells determines cooperation with CD8+ T cells. Immunity.

[125] Audsley K.M., McDonnell A.M., Waithman J. (2020). Cross-Presenting XCR1(+) Dendritic Cells as Targets for Cancer Immunotherapy. Cells.

[126] Thomachot M.C., Bendriss-Vermare N., Massacrier C., Biota C., Treilleux I., Goddard S., Caux C., Bachelot T., Blay J.Y., Menetrier-Caux C. (2004). Breast carcinoma cells promote the differentiation of CD34+ progenitors towards 2 different subpopulations of dendritic cells with CD1a(high)CD86(-)Langerin- and CD1a(+)CD86(+)Langerin+ phenotypes. Int. J. Cancer.

[127] Middel P., Brauneck S., Meyer W., Radzun H.J. (2010). Chemokine-mediated distribution of dendritic cell subsets in renal cell carcinoma. BMC Cancer.

[128] Troy A.J., Summers K.L., Davidson P.J., Atkinson C.H., Hart D.N. (1998). Minimal recruitment and activation of dendritic cells within renal cell carcinoma. Clin. Cancer Res..

[129] Scarpino S., Stoppacciaro A., Ballerini F., Marchesi M., Prat M., Stella M.C., Sozzani S., Allavena P., Mantovani A., Ruco L.P. (2000). Papillary carcinoma of the thyroid: hepatocyte growth factor (HGF) stimulates tumor cells to release chemokines active in recruiting dendritic cells. Am. J. Pathol..

[130] Spranger S., Bao R., Gajewski T.F. (2015). Melanoma-intrinsic beta-catenin signalling prevents anti-tumour immunity. Nature.

[131] Bell D., Chomarat P., Broyles D., Netto G., Harb G.M., Lebecque S., Valladeau J., Davoust J., Palucka K.A., Banchereau J. (1999). In breast carcinoma tissue, immature dendritic cells reside within the tumor, whereas mature dendritic cells are located in peritumoral areas. J. Exp. Med..

[132] Villablanca E.J., Raccosta L., Zhou D., Fontana R., Maggioni D., Negro A., Sanvito F., Ponzoni M., Valentinis B., Bregni M. (2010). Tumor-mediated liver X receptor-alpha activation inhibits CC chemokine receptor-7 expression on dendritic cells and dampens antitumor responses. Nat. Med..

[133] Hubert P., Herman L., Maillard C., Caberg J.H., Nikkels A., Pierard G., Foidart J.M., Noel A., Boniver J., Delvenne P. (2007). Defensins induce the recruitment of dendritic cells in cervical human papillomavirus-associated (pre)neoplastic lesions formed in vitro and transplanted in vivo. FASEB J..

[134] Findlay E.G., Currie A.J., Zhang A., Ovciarikova J., Young L., Stevens H., McHugh B.J., Canel M., Gray M., Milling S.W.F. (2019). Exposure to the antimicrobial peptide LL-37 produces dendritic cells optimized for immunotherapy. Oncoimmunology.

[135] Liang W., Chen K., Gong W., Yoshimura T., Le Y., Wang Y., Wang J.M. (2020). The Contribution of Chemoattractant GPCRs, Formylpeptide Receptors, to Inflammation and Cancer. Front. Endocrinol (Lausanne).

[136] Vacchelli E., Ma Y., Baracco E.E., Sistigu A., Enot D.P., Pietrocola F., Yang H., Adjemian S., Chaba K., Semeraro M. (2015). Chemotherapy-induced antitumor immunity requires formyl peptide receptor 1. Science.

[137] Piao C., Cai L., Qiu S., Jia L., Song W., Du J. (2015). Complement 5a Enhances Hepatic Metastases of Colon Cancer via Monocyte Chemoattractant Protein-1-mediated Inflammatory Cell Infiltration. J. Biol. Chem..

[138] Charles J., Di Domizio J., Salameire D., Bendriss-Vermare N., Aspord C., Muhammad R., Lefebvre C., Plumas J., Leccia M.T., Chaperot L. (2010). Characterization of circulating dendritic cells in melanoma: role of CCR6 in plasmacytoid dendritic cell recruitment to the tumor. J. Investig. Dermatol..

[139] Salio M., Cella M., Vermi W., Facchetti F., Palmowski M.J., Smith C.L., Shepherd D., Colonna M., Cerundolo V. (2003). Plasmacytoid dendritic cells prime IFN-gamma-secreting melanoma-specific CD8 lymphocytes and are found in primary melanoma lesions. Eur. J. Immunol..

[140] Vermi W., Bonecchi R., Facchetti F., Bianchi D., Sozzani S., Festa S., Berenzi A., Cella M., Colonna M. (2003). Recruitment of immature plasmacytoid dendritic cells (plasmacytoid monocytes) and myeloid dendritic cells in primary cutaneous melanomas. J. Pathol..

[141] Zou W., Machelon V., Coulomb-L’Hermin A., Borvak J., Nome F., Isaeva T., Wei S., Krzysiek R., Durand-Gasselin I., Gordon A. (2001). Stromal-derived factor-1 in human tumors recruits and alters the function of plasmacytoid precursor dendritic cells. Nat. Med..

[142] Kryczek I., Lange A., Mottram P., Alvarez X., Cheng P., Hogan M., Moons L., Wei S., Zou L., Machelon V. (2005). CXCL12 and vascular endothelial growth factor synergistically induce neoangiogenesis in human ovarian cancers. Cancer Res..

[143] Vanbervliet B., Bendriss-Vermare N., Massacrier C., Homey B., de Bouteiller O., Briere F., Trinchieri G., Caux C. (2003). The inducible CXCR3 ligands control plasmacytoid dendritic cell responsiveness to the constitutive chemokine stromal cell-derived factor 1 (SDF-1)/CXCL12. J. Exp. Med..

[144] Perrot I., Blanchard D., Freymond N., Isaac S., Guibert B., Pacheco Y., Lebecque S. (2007). Dendritic cells infiltrating human non-small cell lung cancer are blocked at immature stage. J. Immunol..

[145] Qian B.Z., Li J., Zhang H., Kitamura T., Zhang J., Campion L.R., Kaiser E.A., Snyder L.A., Pollard J.W. (2011). CCL2 recruits inflammatory monocytes to facilitate breast-tumour metastasis. Nature.

[146] Veglia F., Gabrilovich D.I. (2017). Dendritic cells in cancer: The role revisited. Curr. Opin. Immunol..

[147] Spary L.K., Salimu J., Webber J.P., Clayton A., Mason M.D., Tabi Z. (2014). Tumor stroma-derived factors skew monocyte to dendritic cell differentiation toward a suppressive CD14(+) PD-L1(+) phenotype in prostate cancer. Oncoimmunology.

[148] Bogunovic D., O’Neill D.W., Belitskaya-Levy I., Vacic V., Yu Y.L., Adams S., Darvishian F., Berman R., Shapiro R., Pavlick A.C. (2009). Immune profile and mitotic index of metastatic melanoma lesions enhance clinical staging in predicting patient survival. Proc. Natl. Acad. Sci. USA.

[149] Hubert M., Gobbini E., Couillault C., Manh T.V., Doffin A.C., Berthet J., Rodriguez C., Ollion V., Kielbassa J., Sajous C. (2020). IFN-III is selectively produced by cDC1 and predicts good clinical outcome in breast cancer. Sci Immunol..

[150] Balan S., Saxena M., Bhardwaj N. (2019). Dendritic cell subsets and locations. Int. Rev. Cell Mol. Biol..

[151] Tran Janco J.M., Lamichhane P., Karyampudi L., Knutson K.L. (2015). Tumor-infiltrating dendritic cells in cancer pathogenesis. J. Immunol..

[152] Hegde S., Krisnawan V.E., Herzog B.H., Zuo C., Breden M.A., Knolhoff B.L., Hogg G.D., Tang J.P., Baer J.M., Mpoy C. (2020). Dendritic Cell Paucity Leads to Dysfunctional Immune Surveillance in Pancreatic Cancer. Cancer Cell.

[153] Goc J., Germain C., Vo-Bourgais T.K., Lupo A., Klein C., Knockaert S., de Chaisemartin L., Ouakrim H., Becht E., Alifano M. (2014). Dendritic cells in tumor-associated tertiary lymphoid structures signal a Th1 cytotoxic immune contexture and license the positive prognostic value of infiltrating CD8+ T cells. Cancer Res..

[154] Mansfield A.S., Heikkila P., von Smitten K., Vakkila J., Leidenius M. (2011). Metastasis to sentinel lymph nodes in breast cancer is associated with maturation arrest of dendritic cells and poor co-localization of dendritic cells and CD8+ T cells. Virchows Arch..

[155] Adhikaree J., Franks H.A., Televantos C., Vaghela P., Kaur A.P., Walker D., Schmitz M., Jackson A.M., Patel P.M. (2019). Impaired circulating myeloid CD1c+ dendritic cell function in human glioblastoma is restored by p38 inhibition—Implications for the next generation of DC vaccines. Oncoimmunology.

[156] Suzuki A., Masuda A., Nagata H., Kameoka S., Kikawada Y., Yamakawa M., Kasajima T. (2002). Mature dendritic cells make clusters with T cells in the invasive margin of colorectal carcinoma. J. Pathol..

[157] Kocian P., Sedivcova M., Drgac J., Cerna K., Hoch J., Kodet R., Bartunkova J., Spisek R., Fialova A. (2011). Tumor-infiltrating lymphocytes and dendritic cells in human colorectal cancer: their relationship to KRAS mutational status and disease recurrence. Hum. Immunol..

[158] Truxova I., Kasikova L., Hensler M., Skapa P., Laco J., Pecen L., Belicova L., Praznovec I., Halaska M.J., Brtnicky T. (2018). Mature dendritic cells correlate with favorable immune infiltrate and improved prognosis in ovarian carcinoma patients. J. Immunother Cancer.

[159] Ladanyi A., Kiss J., Somlai B., Gilde K., Fejos Z., Mohos A., Gaudi I., Timar J. (2007). Density of DC-LAMP(+) mature dendritic cells in combination with activated T lymphocytes infiltrating primary cutaneous melanoma is a strong independent prognostic factor. Cancer Immunol. Immunother..

[160] Conrad C., Gregorio J., Wang Y.H., Ito T., Meller S., Hanabuchi S., Anderson S., Atkinson N., Ramirez P.T., Liu Y.J. (2012). Plasmacytoid dendritic cells promote immunosuppression in ovarian cancer via ICOS costimulation of Foxp3(+) T-regulatory cells. Cancer Res..

[161] Labidi-Galy S.I., Treilleux I., Goddard-Leon S., Combes J.D., Blay J.Y., Ray-Coquard I., Caux C., Bendriss-Vermare N. (2012). Plasmacytoid dendritic cells infiltrating ovarian cancer are associated with poor prognosis. Oncoimmunology.

[162] Gagliostro V., Seeger P., Garrafa E., Salvi V., Bresciani R., Bosisio D., Sozzani S. (2016). Pro-lymphangiogenic properties of IFN-gamma-activated human dendritic cells. Immunol. Lett..

[163] Shi W., Li X., Porter J.L., Ostrodi D.H., Yang B., Li J., Wang Y., Zhang J., Bai L., Jiao S. (2014). Level of plasmacytoid dendritic cells is increased in non-small cell lung carcinoma. Tumour Biol..

[164] Sisirak V., Faget J., Gobert M., Goutagny N., Vey N., Treilleux I., Renaudineau S., Poyet G., Labidi-Galy S.I., Goddard-Leon S. (2012). Impaired IFN-alpha production by plasmacytoid dendritic cells favors regulatory T-cell expansion that may contribute to breast cancer progression. Cancer Res..

[165] Gadalla R., Hassan H., Ibrahim S.A., Abdullah M.S., Gaballah A., Greve B., El-Deeb S., El-Shinawi M., Mohamed M.M. (2019). Tumor microenvironmental plasmacytoid dendritic cells contribute to breast cancer lymph node metastasis via CXCR4/SDF-1 axis. Breast Cancer Res. Treat..

[166] Han N., Zhang Z., Liu S., Ow A., Ruan M., Yang W., Zhang C. (2017). Increased tumor-infiltrating plasmacytoid dendritic cells predicts poor prognosis in oral squamous cell carcinoma. Arch. Oral Biol..

[167] Bakdash G., Buschow S.I., Gorris M.A., Halilovic A., Hato S.V., Skold A.E., Schreibelt G., Sittig S.P., Torensma R., Duiveman-de Boer T. (2016). Expansion of a BDCA1+CD14+ Myeloid Cell Population in Melanoma Patients May Attenuate the Efficacy of Dendritic Cell Vaccines. Cancer Res..

[168] Bol K.F., Schreibelt G., Rabold K., Wculek S.K., Schwarze J.K., Dzionek A., Teijeira A., Kandalaft L.E., Romero P., Coukos G. (2019). The clinical application of cancer immunotherapy based on naturally circulating dendritic cells. J. Immunother. Cancer.

[169] Mastelic-Gavillet B., Balint K., Boudousquie C., Gannon P.O., Kandalaft L.E. (2019). Personalized Dendritic Cell Vaccines-Recent Breakthroughs and Encouraging Clinical Results. Front. Immunol..

[170] Perez C.R., De Palma M. (2019). Engineering dendritic cell vaccines to improve cancer immunotherapy. Nat. Commun..

[171] Wculek S.K., Cueto F.J., Mujal A.M., Melero I., Krummel M.F., Sancho D. (2020). Dendritic cells in cancer immunology and immunotherapy. Nat. Rev. Immunol..

[172] Palucka K., Banchereau J. (2013). Dendritic-cell-based therapeutic cancer vaccines. Immunity.

[173] Saxena M., Balan S., Roudko V., Bhardwaj N. (2018). Towards superior dendritic-cell vaccines for cancer therapy. Nat. Biomed. Eng..

[174] Di Pucchio T., Pilla L., Capone I., Ferrantini M., Montefiore E., Urbani F., Patuzzo R., Pennacchioli E., Santinami M., Cova A. (2006). Immunization of stage IV melanoma patients with Melan-A/MART-1 and gp100 peptides plus IFN-alpha results in the activation of specific CD8(+) T cells and monocyte/dendritic cell precursors. Cancer Res..

[175] Fay J.W., Palucka A.K., Paczesny S., Dhodapkar M., Johnston D.A., Burkeholder S., Ueno H., Banchereau J. (2006). Long-term outcomes in patients with metastatic melanoma vaccinated with melanoma peptide-pulsed CD34(+) progenitor-derived dendritic cells. Cancer Immunol. Immunother..

[176] Kantoff P.W., Higano C.S., Shore N.D., Berger E.R., Small E.J., Penson D.F., Redfern C.H., Ferrari A.C., Dreicer R., Sims R.B. (2010). Sipuleucel-T immunotherapy for castration-resistant prostate cancer. N. Engl. J. Med..

[177] Parmiani G., Pilla L., Castelli C., Rivoltini L. (2003). Vaccination of patients with solid tumours. Ann. Oncol..

[178] Briseno C.G., Haldar M., Kretzer N.M., Wu X., Theisen D.J., Kc W., Durai V., Grajales-Reyes G.E., Iwata A., Bagadia P. (2016). Distinct Transcriptional Programs Control Cross-Priming in Classical and Monocyte-Derived Dendritic Cells. Cell Rep..

[179] Helft J., Bottcher J., Chakravarty P., Zelenay S., Huotari J., Schraml B.U., Goubau D., Reis e Sousa C. (2015). GM-CSF Mouse Bone Marrow Cultures Comprise a Heterogeneous Population of CD11c(+)MHCII(+) Macrophages and Dendritic Cells. Immunity.

[180] Carreno B.M., Becker-Hapak M., Huang A., Chan M., Alyasiry A., Lie W.R., Aft R.L., Cornelius L.A., Trinkaus K.M., Linette G.P. (2013). IL-12p70-producing patient DC vaccine elicits Tc1-polarized immunity. J. Clin. Investig..

[181] Anguille S., Smits E.L., Lion E., van Tendeloo V.F., Berneman Z.N. (2014). Clinical use of dendritic cells for cancer therapy. Lancet Oncol.

[182] Balan S., Ollion V., Colletti N., Chelbi R., Montanana-Sanchis F., Liu H., Vu Manh T.P., Sanchez C., Savoret J., Perrot I. (2014). Human XCR1+ dendritic cells derived in vitro from CD34+ progenitors closely resemble blood dendritic cells, including their adjuvant responsiveness, contrary to monocyte-derived dendritic cells. J. Immunol..

[183] De Vries I.J., Krooshoop D.J., Scharenborg N.M., Lesterhuis W.J., Diepstra J.H., Van Muijen G.N., Strijk S.P., Ruers T.J., Boerman O.C., Oyen W.J. (2003). Effective migration of antigen-pulsed dendritic cells to lymph nodes in melanoma patients is determined by their maturation state. Cancer Res..

[184] Shinde P., Fernandes S., Melinkeri S., Kale V., Limaye L. (2018). Compromised functionality of monocyte-derived dendritic cells in multiple myeloma patients may limit their use in cancer immunotherapy. Sci. Rep..

[185] Steinman R.M. (2008). Dendritic cells in vivo: a key target for a new vaccine science. Immunity.

[186] Breton G., Lee J., Zhou Y.J., Schreiber J.J., Keler T., Puhr S., Anandasabapathy N., Schlesinger S., Caskey M., Liu K. (2015). Circulating precursors of human CD1c+ and CD141+ dendritic cells. J. Exp. Med..

[187] Tel J., Aarntzen E.H., Baba T., Schreibelt G., Schulte B.M., Benitez-Ribas D., Boerman O.C., Croockewit S., Oyen W.J., van Rossum M. (2013). Natural human plasmacytoid dendritic cells induce antigen-specific T-cell responses in melanoma patients. Cancer Res..

[188] Schreibelt G., Bol K.F., Westdorp H., Wimmers F., Aarntzen E.H., Duiveman-de Boer T., van de Rakt M.W., Scharenborg N.M., de Boer A.J., Pots J.M. (2016). Effective Clinical Responses in Metastatic Melanoma Patients after Vaccination with Primary Myeloid Dendritic Cells. Clin. Cancer Res..

[189] Prue R.L., Vari F., Radford K.J., Tong H., Hardy M.Y., D’Rozario R., Waterhouse N.J., Rossetti T., Coleman R., Tracey C. (2015). A phase I clinical trial of CD1c (BDCA-1)+ dendritic cells pulsed with HLA-A*0201 peptides for immunotherapy of metastatic hormone refractory prostate cancer. J. Immunother..

[190] Kirkling M.E., Cytlak U., Lau C.M., Lewis K.L., Resteu A., Khodadadi-Jamayran A., Siebel C.W., Salmon H., Merad M., Tsirigos A. (2018). Notch Signaling Facilitates In Vitro Generation of Cross-Presenting Classical Dendritic Cells. Cell Rep..

[191] Balan S., Arnold-Schrauf C., Abbas A., Couespel N., Savoret J., Imperatore F., Villani A.C., Vu Manh T.P., Bhardwaj N., Dalod M. (2018). Large-Scale Human Dendritic Cell Differentiation Revealing Notch-Dependent Lineage Bifurcation and Heterogeneity. Cell Rep..

[192] Rosa F.F., Pires C.F., Kurochkin I., Ferreira A.G., Gomes A.M., Palma L.G., Shaiv K., Solanas L., Azenha C., Papatsenko D. (2018). Direct reprogramming of fibroblasts into antigen-presenting dendritic cells. Sci. Immunol..

[193] Wculek S.K., Amores-Iniesta J., Conde-Garrosa R., Khouili S.C., Melero I., Sancho D. (2019). Effective cancer immunotherapy by natural mouse conventional type-1 dendritic cells bearing dead tumor antigen. J. Immunother. Cancer.

[194] Pearson F.E., Chang K., Minoda Y., Rojas I.M.L., Haigh O.L., Daraj G., Tullett K.M., Radford K.J. (2018). Activation of human CD141(+) and CD1c(+) dendritic cells in vivo with combined TLR3 and TLR7/8 ligation. Immunol. Cell Biol..

[195] Roselli E., Araya P., Nunez N.G., Gatti G., Graziano F., Sedlik C., Benaroch P., Piaggio E., Maccioni M. (2019). TLR3 Activation of Intratumoral CD103(+) Dendritic Cells Modifies the Tumor Infiltrate Conferring Anti-tumor Immunity. Front. Immunol..

[196] Tanyi J.L., Bobisse S., Ophir E., Tuyaerts S., Roberti A., Genolet R., Baumgartner P., Stevenson B.J., Iseli C., Dangaj D. (2018). Personalized cancer vaccine effectively mobilizes antitumor T cell immunity in ovarian cancer. Sci. Trans. Med..

[197] Squadrito M.L., Cianciaruso C., Hansen S.K., De Palma M. (2018). EVIR: chimeric receptors that enhance dendritic cell cross-dressing with tumor antigens. Nat. Methods.

[198] Han D., Liu J., Chen C., Dong L., Liu Y., Chang R., Huang X., Liu Y., Wang J., Dougherty U. (2019). Anti-tumour immunity controlled through mRNA m(6)A methylation and YTHDF1 in dendritic cells. Nature.

[199] Hobo W., Maas F., Adisty N., de Witte T., Schaap N., van der Voort R., Dolstra H. (2010). siRNA silencing of PD-L1 and PD-L2 on dendritic cells augments expansion and function of minor histocompatibility antigen-specific CD8+ T cells. Blood.

[200] Chow M.T., Ozga A.J., Servis R.L., Frederick D.T., Lo J.A., Fisher D.E., Freeman G.J., Boland G.M., Luster A.D. (2019). Intratumoral Activity of the CXCR3 Chemokine System Is Required for the Efficacy of Anti-PD-1 Therapy. Immunity.

[201] Garris C.S., Arlauckas S.P., Kohler R.H., Trefny M.P., Garren S., Piot C., Engblom C., Pfirschke C., Siwicki M., Gungabeesoon J. (2018). Successful Anti-PD-1 Cancer Immunotherapy Requires T Cell-Dendritic Cell Crosstalk Involving the Cytokines IFN-gamma and IL-12. Immunity.

[202] Tullett K.M., Lahoud M.H., Radford K.J. (2014). Harnessing Human Cross-Presenting CLEC9A(+)XCR1(+) Dendritic Cells for Immunotherapy. Front. Immunol..

[203] Cauwels A., Van Lint S., Paul F., Garcin G., De Koker S., Van Parys A., Wueest T., Gerlo S., Van der Heyden J., Bordat Y. (2018). Delivering Type I Interferon to Dendritic Cells Empowers Tumor Eradication and Immune Combination Treatments. Cancer Res..

[204] Okada N., Mori N., Koretomo R., Okada Y., Nakayama T., Yoshie O., Mizuguchi H., Hayakawa T., Nakagawa S., Mayumi T. (2005). Augmentation of the migratory ability of DC-based vaccine into regional lymph nodes by efficient CCR7 gene transduction. Gene Ther..

[205] Yang X., Lian K., Meng T., Liu X., Miao J., Tan Y., Yuan H., Hu F. (2018). Immune Adjuvant Targeting Micelles Allow Efficient Dendritic Cell Migration to Lymph Nodes for Enhanced Cellular Immunity. ACS Appl. Mater. Interfaces.

[206] Moran T.P., Nakano H., Kondilis-Mangum H.D., Wade P.A., Cook D.N. (2014). Epigenetic control of Ccr7 expression in distinct lineages of lung dendritic cells. J. Immunol..

[207] Lee J.M., Lee M.H., Garon E., Goldman J.W., Salehi-Rad R., Baratelli F.E., Schaue D., Wang G., Rosen F., Yanagawa J. (2017). Phase I Trial of Intratumoral Injection of CCL21 Gene-Modified Dendritic Cells in Lung Cancer Elicits Tumor-Specific Immune Responses and CD8(+) T-cell Infiltration. Clin. Cancer Res..

[208] Terhorst D., Fossum E., Baranska A., Tamoutounour S., Malosse C., Garbani M., Braun R., Lechat E., Crameri R., Bogen B. (2015). Laser-assisted intradermal delivery of adjuvant-free vaccines targeting XCR1+ dendritic cells induces potent antitumoral responses. J. Immunol..

[209] Hartung E., Becker M., Bachem A., Reeg N., Jakel A., Hutloff A., Weber H., Weise C., Giesecke C., Henn V. (2015). Induction of potent CD8 T cell cytotoxicity by specific targeting of antigen to cross-presenting dendritic cells in vivo via murine or human XCR1. J. Immunol..

[210] Sanchez-Paulete A.R., Teijeira A., Quetglas J.I., Rodriguez-Ruiz M.E., Sanchez-Arraez A., Labiano S., Etxeberria I., Azpilikueta A., Bolanos E., Ballesteros-Briones M.C. (2018). Intratumoral Immunotherapy with XCL1 and sFlt3L Encoded in Recombinant Semliki Forest Virus-Derived Vectors Fosters Dendritic Cell-Mediated T-cell Cross-Priming. Cancer Res..

[211] Vanpouille-Box C., Alard A., Aryankalayil M.J., Sarfraz Y., Diamond J.M., Schneider R.J., Inghirami G., Coleman C.N., Formenti S.C., Demaria S. (2017). DNA exonuclease Trex1 regulates radiotherapy-induced tumour immunogenicity. Nat. Commun..

